# Pharmacokinetics of a single inhalation of hydrogen gas in pigs

**DOI:** 10.1371/journal.pone.0234626

**Published:** 2020-06-19

**Authors:** Motoaki Sano, Genki Ichihara, Yoshinori Katsumata, Takahiro Hiraide, Akeo Hirai, Mizuki Momoi, Tomoyoshi Tamura, Shigeo Ohata, Eiji Kobayashi

**Affiliations:** 1 Department of Cardiology, Keio University School of Medicine, Shinjuku-ku, Tokyo, Japan; 2 Center for Molecular Hydrogen Medicine, Keio University, Minato-ku, Tokyo, Japan; 3 Department of Emergency and Critical Care Medicine, Keio University, Shinjuku-ku, Tokyo, Japan; 4 Department of Organ Fabrication, Keio University School of Medicine, Shinjuku-ku, Tokyo, Japan; 5 Department of Neurology, Juntendo University Graduate School of Medicine, Bunkyo-ku, Tokyo, Japan; Niigata Daigaku, JAPAN

## Abstract

The benefits of inhaling hydrogen gas (H_2_) have been widely reported but its pharmacokinetics have not yet been sufficiently analyzed. We developed a new experimental system in pigs to closely evaluate the process by which H_2_ is absorbed in the lungs, enters the bloodstream, and is distributed, metabolized, and excreted. We inserted and secured catheters into the carotid artery (CA), portal vein (PV), and supra-hepatic inferior vena cava (IVC) to allow repeated blood sampling and performed bilateral thoracotomy to collapse the lungs. Then, using a hydrogen-absorbing alloy canister, we filled the lungs to the maximum inspiratory level with 100% H_2_. The pig was maintained for 30 seconds without resuming breathing, as if they were holding their breath. We collected blood from the three intravascular catheters after 0, 3, 10, 30, and 60 minutes and measured H_2_ concentration by gas chromatography. H_2_ concentration in the CA peaked immediately after breath holding; 3 min later, it dropped to 1/40 of the peak value. Peak H_2_ concentrations in the PV and IVC were 40% and 14% of that in the CA, respectively. However, H_2_ concentration decay in the PV and IVC (half-life: 310 s and 350 s, respectively) was slower than in the CA (half-life: 92 s). At 10 min, H_2_ concentration was significantly higher in venous blood than in arterial blood. At 60 min, H_2_ was detected in the portal blood at a concentration of 6.9–53 nL/mL higher than at steady state, and in the SVC 14–29 nL/mL higher than at steady state. In contrast, H_2_ concentration in the CA decreased to steady state levels. This is the first report showing that inhaled H_2_ is transported to the whole body by advection diffusion and metabolized dynamically.

## Introduction

Inhalation of H_2_ is reported to have beneficial effects in living organisms [1, 2], and clinical trials have demonstrated its efficacy and safety in patients with acute myocardial infarction [3] and post-resuscitation cardiac arrest [4, 5]. On March 3, 2020, the Chinese National Health and Medical Commission recommended “conditional treatment with hydrogen and oxygen inhalation” in addition to the general oxygen therapy measures in the treatment section of the Diagnosis and Treatment Protocol for Novel Coronavirus Pneumonia (Trial Version 7), in accordance with a recommendation notification by the Chinese Non-government Medical Institutions Association [6]. However, the kinetics of inhaled H_2_ in the body have not been sufficiently analyzed to date.

We previously measured, in rats, the time course of H_2_ levels in different tissues after continuous H_2_ inhalation, by inserting a needle-type sensor electrode directly into the tissues [7, 8]. However, since the response of the needle-type hydrogen sensor electrode is slow, this makes it unsuitable for measuring short-term changes in H_2_ concentration in tissues.

In a non-clinical pharmacokinetic study, the distribution of a test drug to various organs and tissues after a single or repeated dose and its change over time should be investigated. In the case of gas, unlike oral and injectable drugs, a non-clinical pharmacokinetic study with a single dose has not been performed. This was because there was no animal protocol for a single-dose study of the gas. The same is true for H_2_. It remained undetermine whether H_2_ diffused from the lungs in a blood flow-independent manner or whether H_2_ was transported throughout the body in a blood flow-dependent manner. Therefore, in the present study, we devised an animal protocol for single-dose inhalation of gas and proved the latter to be true for the first time.

The most effective way of taking H_2_ into circulating blood after a single inhalation is by fully exhaling, then inhaling 100% H_2_ to the maximum inspiration position, and holding your breath for as long as you can endure. In the present study, we examine the pharmacokinetics of H_2_ by replicating this single inhalation method in pigs.

## Materials and methods

### Animals

The present study was designed according to the principles of the ARRIVE (Animal Research: Reporting of In Vivo Experiments) guidelines [9]. Experiments were performed in accordance with the institutional guidelines and the Japanese law on the protection and management of animals. The full ethical proposal was approved by the Research Council and Animal Care and Use Committee of Keio University [approval no: 12094-(7)].

Two female pigs, weighing 22.4 kg and 22.0 kg, were housed in separate cages under temperature- and light-controlled conditions (12-h light/dark cycle) and provided with food and water ad libitum. The pigs were fasted for 12 h prior to surgery, with free access to water. Sedation with medetomidine (0.02 mg/kg) and midazolam (0.1 mg/kg) was followed by endotracheal intubation and mechanical ventilation. Anesthesia was maintained with inhalational isoflurane. Surgery was performed by a surgeon with experience of more than 200 clinical transplant operations, who is a steering member of the transplantation society and a permanent director of the transplantation society of Japan (E.K.).

### Catheter insertion

Before insertion, a catheter (Argyle Medecut LCV-UK kit, 16 GX, 70 cm) was filled with heparinized saline, and the blood collection site was equipped with a three-way stopcock (TERUMO terfusion three-way stopcock, R type). Once at a sufficient depth of anesthesia, the pig was placed in the supine position. A vertical incision of about 10 cm was made in the right side of the neck to expose about 3 cm of the right external jugular vein and the right internal carotid artery (CA). The peripheral side of the right internal CA was ligated with a 1–0 silk thread, a bulldog clip was applied to the medial side, and after An incision was made in CA, a catheter was advanced through the artery to about 5 cm and secured. Subsequently, another prepared catheter was inserted through the right external jugular vein and advanced approximately 25 cm toward the upper hepatic inferior vena cava and fixed. After confirming sufficient reflux blood could be obtained from both catheters, the skin incision was continuously sutured with 5–0 nylon thread.

Next, a midline incision of the upper abdomen was made about 30 cm below the xiphoid process, and the abdomen was opened. The intestinal tract was held to the left to expose the hepatic portal region. An incision was made in the pancreatic vein and a catheter was inserted about 3 cm toward the hepatic portal. The midline incision was closed by continuous suture with 5–0 nylon thread.

### Protocol for achieving a single inhalation of H_2_

A median sternum incision was made from the xiphoid process toward the head. At the end of an expiration, the ventilator was removed to stop the animal breathing, and both lungs were manually compressed to mimic maximal forced exhalation by eliminating residual air.

**Fig 1:**
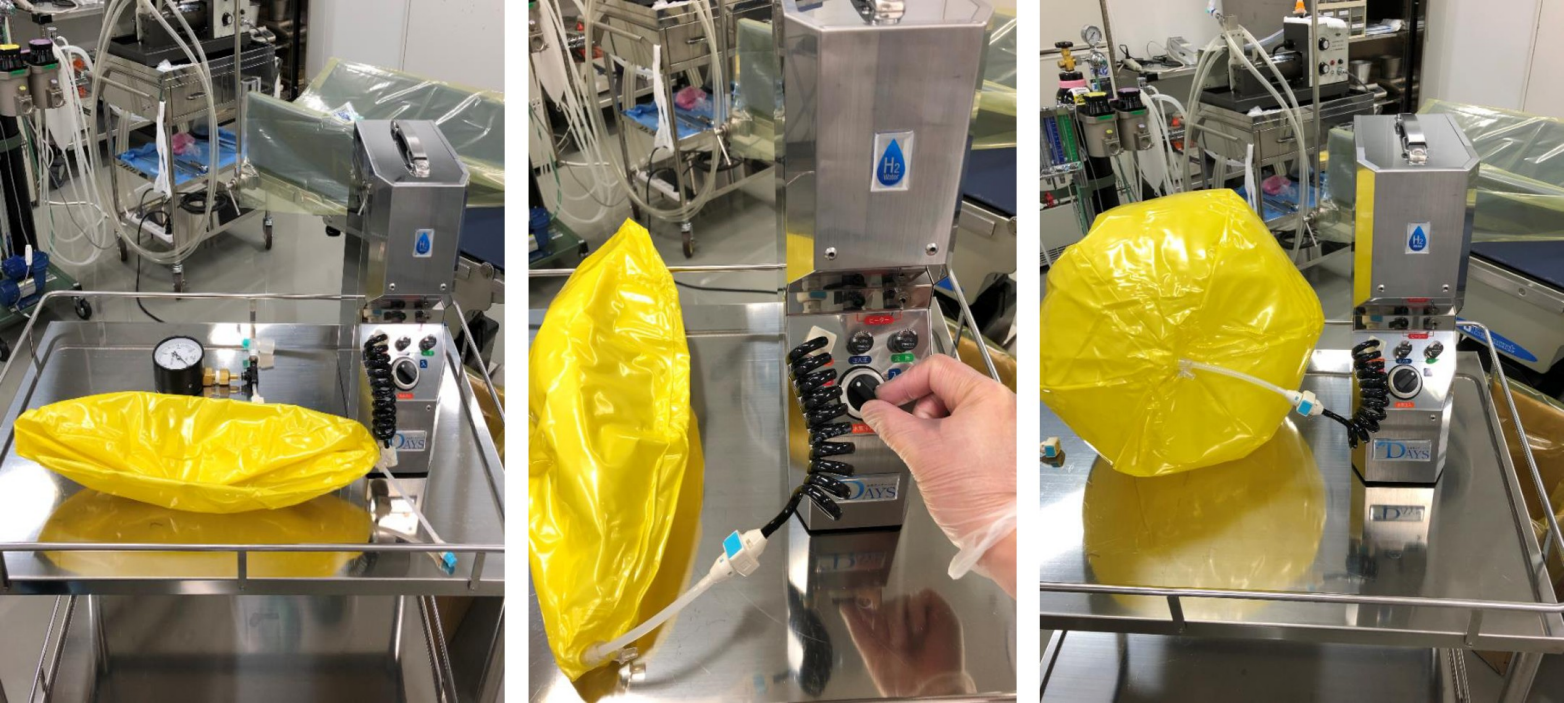
Beach ball being filled with 100% H_2_. The ball is expanding with H_2_ released from a hydrogen-absorbing alloy in an H_2_ filling device.

A beach ball was filled with 100% H_2_ using a hydrogen gas filling device from DAYS (Doctorsman Co., Ltd.) containing hydrogen-absorbing alloys [DAYS (Doctorsman Co., Ltd.)] [10] (**Fig 1**). The device contained a coupler consisting of a plug and socket with a built-in valve, so when the plug and socket were separated, the inflow of air into the beach ball was completely blocked. The beach ball was connected to a tracheal tube then squeezed, using both hands, at a pressure of about 20 mmHg, to fill the lungs with 100% H_2_. This reflected the manner in which a bag valve mask would be pressed. The H_2_-filled lungs were kept at maximal inspiration for approximately 30 s before the tracheal tube was connected to the ventilator and breathing was resumed.

### Blood sampling for H_2_ concentration measurement

Blood was collected from the three intravascular catheters. First, blood was collected in the steady-state condition (before the breath hold, with the chest open). Next, two sets of experiments were performed per pig. In the first set, blood was collected immediately after the breath hold and at 3, 10, 30, and 60 min after restarting ventilation. In the second set, blood was collected immediately after the breath hold and at 3 and 10 min after restarting ventilation.

### Measurement of H_2_ concentration

A needle was inserted into the rubber lid of a 13.5-mL sealed vial, 1 mL of air was extracted, and 1 mL of blood was injected. To prevent outgassing, wax was immediately applied to the rubber lid and the injected hole was sealed.

H_2_ in the blood was released into the air phase in the closed vial. Some of the air phase (0.2 mL, 0.4 mL or 1 mL, depending on the H_2_ concentration) was collected from the vial and H_2_ concentration was measured by gas chromatography (TRIlyzer mBA-3000, Taiyo, Co., Ltd. Osaka, Japan). A calibration curve was obtained using standard H_2_ gas of 0, 5, 50 and 130 ppm. Each sample was measured twice. The concentration of the sample taken before H_2_ inhalation was subtracted as background.

### Statistical analysis

Data are expressed as the mean ± standard error of the mean. One-way analysis of variance followed by a Tukey–Kramer multiple comparisons test was used to compare the H_2_ concentrations between measurement sites. *P* < 0.05 was considered significant. All data were analyzed using GraphPad Prism 8.4 (GraphPad Software Inc., La Jolla, CA).

## Results

### H_2_ concentration in circulating blood at steady state

Mammalian cells do not produce H_2_ as they lack the hydrogenase activity necessary for its formation. Instead, resident bacteria in the colon produce a considerable amount of H_2_ via anaerobic fermentation of unabsorbed carbohydrates. It is generally assumed that H_2_ produced by bacterial fermentation in the colon is transferred to the portal circulation and excreted through the breath. In a previous breath gas analysis we conducted in healthy volunteers, we found that the concentration of H_2_ in the breath varies widely (1–56 ppm) between individuals [11].

In the present experiment, we detected minimal H_2_ in the carotid artery (CA) in the steady-state condition in both pigs. In contrast, H_2_ concentration in the portal vein (PV) was 67 nL/mL and 8.8 nL/mL for the first and second pigs, respectively, and in the supra-hepatic inferior vena cava (IVC) it was 18 nL/mL and 1.9 nL/mL, respectively. We expect that the large difference between the two pigs in PV H_2_ concentration is due to differences in H_2_ production ability by colonic bacteria.

These results indicate that H_2_ produced by bacteria in the colon is carried by the portal circulation, most of it is trapped in the liver, and the remaining H_2_ is excreted from the lungs.

### Pharmacokinetics a single inhalation of H_2_

In the first set of experiments, blood H_2_ concentration was tracked until 60 min after breathing was resumed. After that, breathing was stopped again at the end of an exhalation and the lungs were manually compressed to reduce residual air, and then expanded with 100% H_2_ for the second set of experiments. In the second set, H_2_ concentration in the circulating blood was monitored for 10 min.

**Fig 2:**
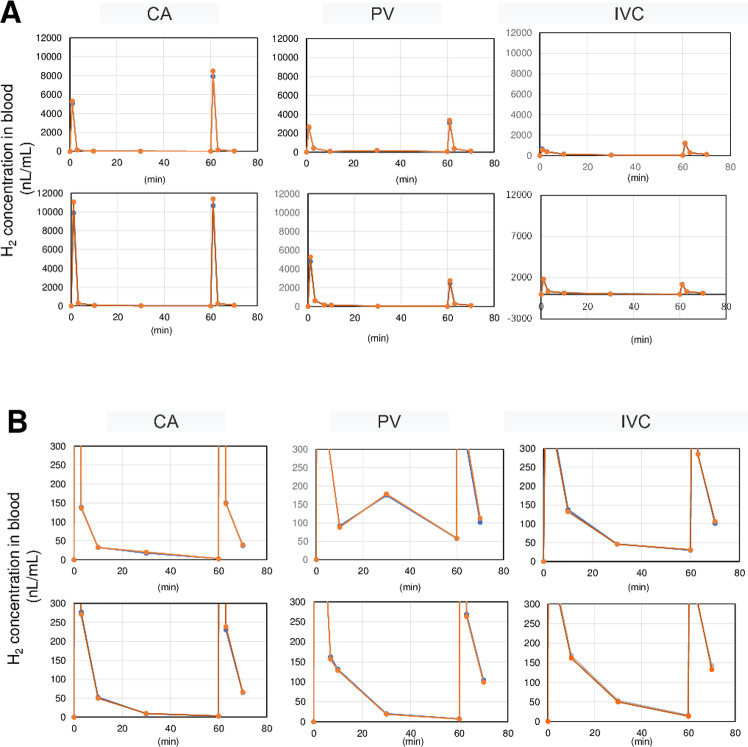
Time course of blood concentration of H_2_ after a single inhalation. (A) Peak H_2_ concentration in the CA reached 28–60% saturation. Peak H_2_ concentration in venous blood (PV, IVC) was much lower than that in arterial blood (CA>>PV>IVC). (B) Enlarged low-concentration areas from Fig 3A. After 10 min, blood H_2_ concentrations were highest in the IVC, then the PV, and lowest in the CA. After 60 min, H_2_ concentration in the PV and IVC remained higher than at steady state. In A and B, upper and lower graphs in each panel show readings from pig 1 and pig 2, respectively. Duplicate H_2_ concentration measurements are overlaid. CA, carotid artery; PV, portal vein; IVC, supra-hepatic inferior vena cava.

Immediately after the end of the breath hold, the peak H_2_ concentration in the CA of the first pig was 5000 nL/mL in the first set of experiments and 7900 nL/mL in the second set. In the second pig, the concentrations were 10000 and 11000 nL/mL, respectively. (**Fig 2A**). H_2_ concentration of a 100% aqueous solution was 17,600 nL/mL, meaning that peak H_2_ in the CA reached 28–60% saturation by this inhalation method. Peak H_2_ concentration in venous blood (PV, IVC) was much lower than that in arterial blood (CA>>PV>IVC). This indicates that H_2_ is not simply diffused, but diffuses while being carried by the bloodstream (advection diffusion), and most H_2_ is consumed by the tissues.

**Fig 3:**
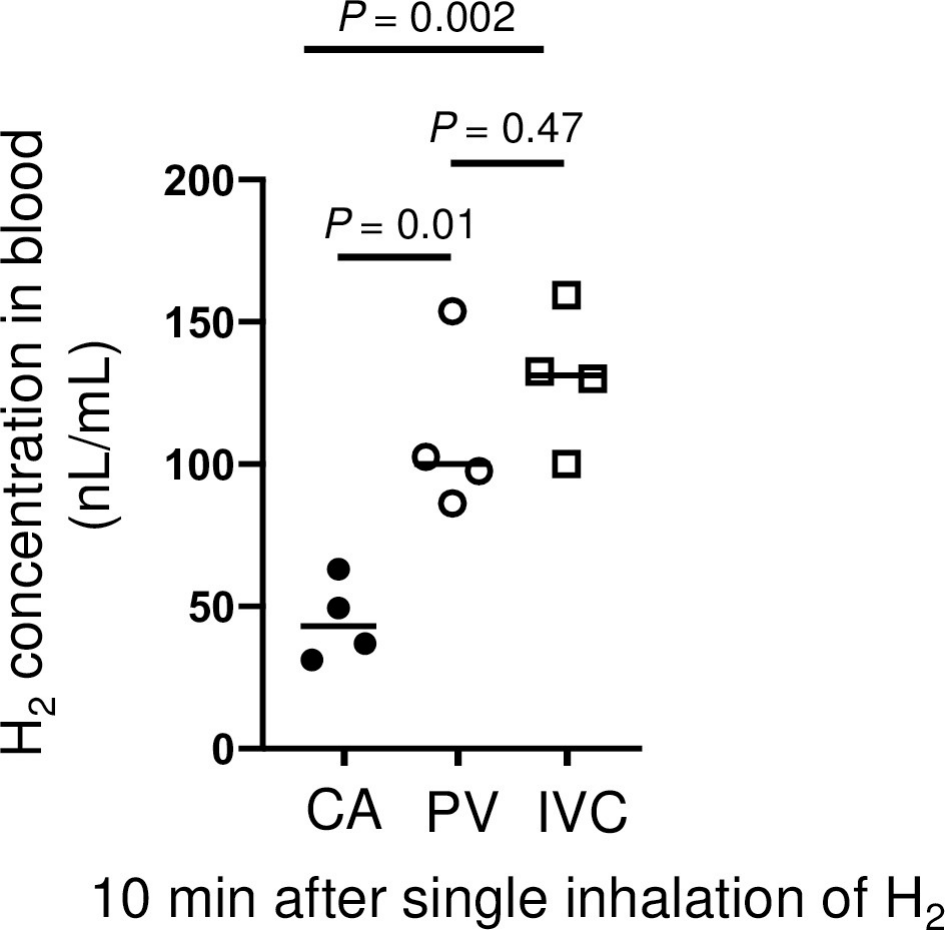
Blood concentration of H_2_ 10 min after a single inhalation. Venous H_2_ concentrations (PV, IVC) were significantly higher than arterial H_2_ concentrations (CA). Individual readings (two readings from two animals from each intravascular catheter) and means are shown.

**Fig 4:**
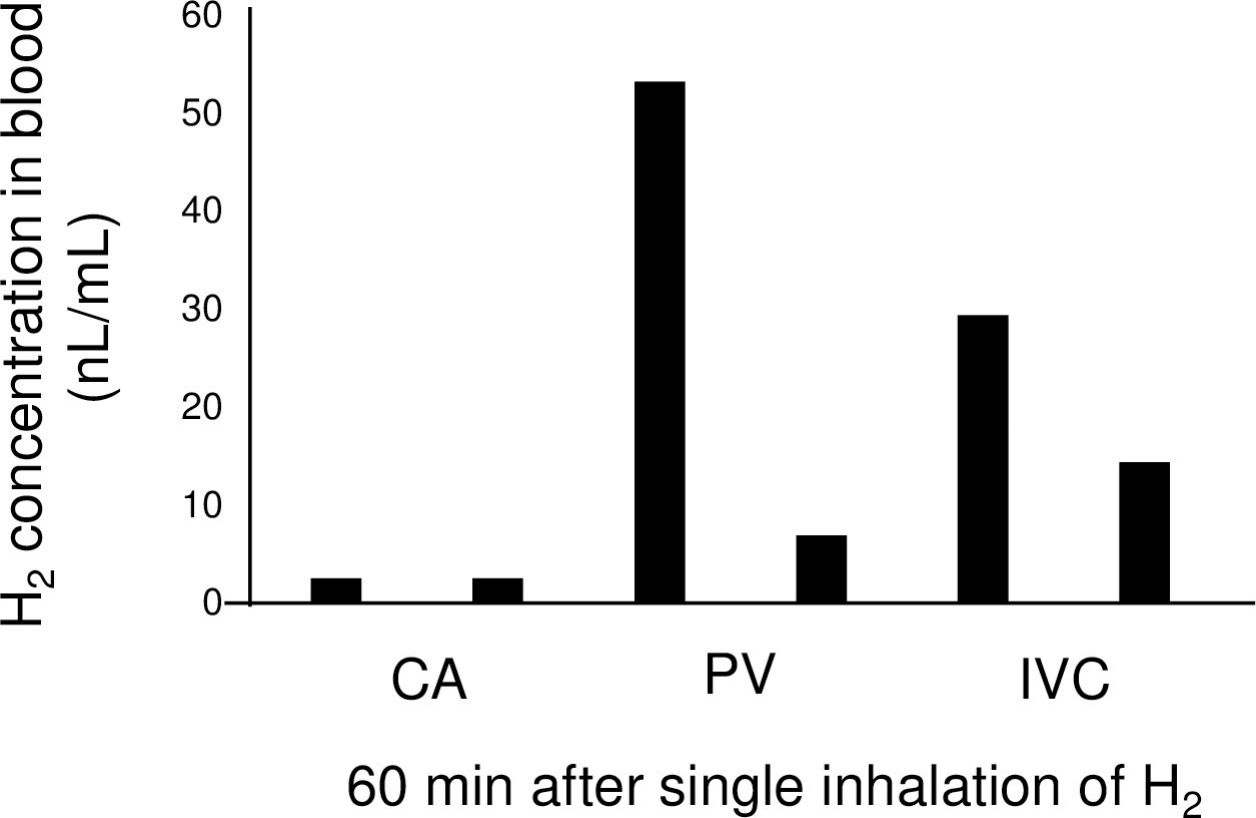
Blood concentration of H_2_ 60 min after a single inhalation. By 60 min, H_2_ had almost disappeared in the CA but remained in the IVC and PV. Data are one reading per animal from each intravascular catheter.

H_2_ concentration decreases rapidly in arterial blood (half-life: 92 s) but more slowly in venous blood (half-life: PV, 310 s; IVC, 350 s) (**Fig 2B**). Consequently, H_2_ concentration after 10 min was greatest in the IVC, then in the PV, and lowest in the CA (**Fig 3**)—the opposite to that at peak concentration. At 60 min after resuming breathing, H_2_ in the CA had almost disappeared (2.5 nL/mL) (**Fig 4**), but higher concentrations of H_2_ were detected in the PV and IVC than at steady state. These results indicate that H_2_ is absorbed in the tissues, then gradually exits the tissues and returns to the heart via venous flow. In other words, a considerable amount of H_2_ remains in the tissues throughout the body even 60 min after inhalation of H_2_.

## Discussion

This is the first preclinical study to investigate the kinetics of single-dose inhalation of H_2_ in the body. We devised a protocol that allows pigs to inhale H_2_ only once. The ventilator was removed from the intubated pig at the end of expiration. Both lungs were compressed by hand to release the remaining air. We defined this state as the estimated position at maximum exhalation. A beach ball filled with 100% H_2_ was connected to a tracheal tube and H_2_ was pumped into the lungs until inflated to the estimated position of maximal inspiration by squeezing the beach ball with both arms. We kept the H_2_-inflated lungs intact for a while. We modeled the behavior of holding the breath after inhaling as much H_2_ as possible using this series of methods.

Since many animal experiments [12–14] and clinical studies [4, 5, 15] have been conducted to examine the protective effect of H_2_ inhalation on the brain, the CA was chosen as the first blood collection point to prove that the inhaled H_2_ can reach the brain efficiently. The liver has a dual blood supply from the PV and the hepatic artery. About 75% of the blood flow to the liver comes from the PV and 25% from the hepatic artery. Oxygen is supplied by the PV and the hepatic artery in half each. Therefore, in contrast to the brain, the liver has been regarded as the organ where inhaled H_2_ is least likely to reach [16]. The liver is the largest organ in the body, performing a number of functions that are essential for life, such as metabolism, detoxification, and excretion, so protecting it with H_2_ is considered to be a great advantage. We wanted to find out how much H_2_ is consumed as it passes through the liver, so we compared the H_2_ concentrations in the PV and supra-hepatic IVC. The H_2_ concentration of CA immediately after inhalation was very high, and it was confirmed that the inhaled H_2_ reached the brain efficiently. The peak H_2_ concentrations of PV and IVC were 40% and 14% of CA, respectively, indicating that inhaled H_2_ is relatively difficult to reach the liver, but the liver actively consumes the H_2_.

H_2_ circulates throughout the body, with only about 10% returning to the venous blood. The arterial blood H_2_ concentration drops rapidly and has a half-life of about 90 seconds. On the other hand, the half-life of venous blood H_2_ concentrations is longer, 310 seconds for PV and 350 seconds for IVC; therefore, 3 minutes after inhalation, H_2_ concentrations in venous blood exceed those in arterial blood. This is presumed to be due to the fact that the H_2_ diffused into the tissues, which was not metabolized, is gradually returned to the venous blood. H_2_ can still be detected in venous blood an hour after a single inhalation, but H_2_ is almost undetectable in arterial blood, perhaps because it is discarded from the lungs.

Gaseous molecules, such as oxygen, carbon dioxide, nitric oxide, and hydrogen sulfide, can bind to the ferrous heme of a variety of proteins with high affinity; thus, they bind to hemoglobin. H_2_, however, does not bind to heme, and its receptor molecules and their downstream effectors have not yet been identified. Inhaled H_2_ is simply dissolved in the plasma and transported to the whole body. Supply of H_2_ via the arterial blood to the tissues depends on blood flow. However, unlike for oxygen, there is no system that keeps H_2_ concentrated in the blood vessels, so it diffuses out of the blood vessels as it travels.

Whether H_2_ is inhaled or drunk in water enriched with dissolved H_2_ [17, 18], breath analysis shows that 60% is excreted in the breath, with 40% being consumed by the body. The amount of H_2_ released from the body surface is estimated to be extremely small—about 0.1% [17]. By comparing the H_2_ concentration in the PV and IVC, we estimate that 64% of H_2_ in the portal blood is trapped just by passing through the liver. Together, these results indicate that H_2_ is consumed by the body, but the molecular mechanism of how H_2_ is metabolized remains unknown.

We, at the Center for Molecular Hydrogen Medicine at Keio University in Tokyo, have demonstrated the therapeutic effects of H_2_ on diseases such as acute myocardial infarction [3, 7], post-cardiac arrest syndrome [4, 5, 13, 14], hemorrhagic shock [19, 20], and organ transplantation [10] in both animal experiments and clinical studies. In patients with severe COVID-19, the immune over-response causes the production of large amounts of cytokines by alveolar macrophages, which becomes a cytokine storm, resulting in the progression of acute respiratory distress syndrome, abnormal blood coagulation, and multiple organ failure [21]. H_2_ gas not only inhibits the overproduction of cytokines [13], but also suppresses vascular endothelial damage [20], facilitates the flow of red blood cells in microvessels and increases the efficiency of gas exchange (M.S. unpublished observation). Accordingly, H_2_ inhalation therapy has great potential to improve the life expectancy of intubated COVID-19 patients admitted to the intensive care unit with severe hypoxemia. The HYBRID II Trial (Efficacy of inhaled HYdrogen on neurological outcome following BRain Ischemia During out-of-hospital cardiac arrest), a multicenter, randomized, double-blind, placebo-controlled, controlled clinical trial investigating the efficacy of H_2_ inhalation therapy for patients after out-of-hospital cardiac arrest, has been underway since 2017 using a hydrogenated oxygen supply device jointly developed by Keio University and Taiyo Nippon Sanso (jRCTs031180352) [4]. In the HYBRID II trial, patients after cardiopulmonary arrest and resuscitation have been treated with hydrogenated oxygen for 18 hours in combination with conventional cooling methods. Prior to Hybrid II, we had conducted an open-label, single-arm, prospective interventional trial at Keio University Hospital in Tokyo in 2014 to evaluate the feasibility and safety of H_2_ inhalation in patients with out-of-hospital cardiac arrest achieved a spontaneous return of circulation [5]. Non-cardiogenic cardiac arrest patients were also enrolled in this TRIAL; 5 patients were entered, one of whom was a CA patient due to severe pneumonia and septic shock. This patient had a stable respiratory state during H_2_ inhalation, but died after H_2_ inhalation was completed due to a rapid deterioration of the respiratory state. Based on this experience, we are considering a study protocol in which patients with severe COVID-19 disease will continue to receive hydrogenated oxygen for at least one week until their hypoxemia has sufficiently improved. In China, the hydrogen-oxygen mixture gas inhaler was certified as a national class III medical device in February, and it is reported to have a certain effect on the improvement of hypoxic symptoms of COVID-19-related pneumonia. We hope to bring this new treatment to patients as quickly as possible in order to save the lives of severely ill patients with COVID-19.

## Conclusion

We developed a pig model in which we could study the pharmacokinetics of a single inhalation of H_2_. Inhaled H_2_ was transported to the whole body by advection diffusion, and metabolized dynamically. The present results will contribute to the knowledge on H_2_ biology that is increasingly being applied to medicine.
